# Novel Immunotherapies for Autoimmune Hepatitis

**DOI:** 10.3389/fped.2017.00008

**Published:** 2017-01-26

**Authors:** Shamir Cassim, Marc Bilodeau, Catherine Vincent, Pascal Lapierre

**Affiliations:** ^1^Laboratoire d’hépatologie cellulaire, Centre de recherche du Centre hospitalier de l’Université de Montréal (CRCHUM), Montréal, QC, Canada; ^2^Département de médecine, Université de Montréal, Montréal, QC, Canada

**Keywords:** treatment, monoclonal antibodies, autoimmune disease, liver, regulatory T cells

## Abstract

Autoimmune hepatitis (AIH) is a multifactorial autoimmune disease of unknown pathogenesis, characterized by a loss of immunological tolerance against liver autoantigens resulting in the progressive destruction of the hepatic parenchyma. Current treatments are based on non-specific immunosuppressive drugs. Although tremendous progress has been made using specific biological agents in other inflammatory diseases, progress has been slow to come for AIH patients. While current treatments are successful in the majority of patients, treatment discontinuation is difficult to achieve, and relapses are frequent. Lifelong immunosuppression is not without risks, especially in the pediatric population; 4% of patient with type 1 AIH will eventually develop hepatocellular carcinoma with a 2.9% probability after 10 years of treatment. Therefore, future treatments should aim to restore tolerance to hepatic autoantigens and induce long-term remission. Promising new immunotherapies have been tested in experimental models of AIH including T and B cell depletion and regulatory CD4^+^ T cells infusion. Clinical studies on limited numbers of patients have also shown encouraging results using B-cell-depleting (rituximab) and anti-TNF-α (infliximab) antibodies. A better understanding of key molecular targets in AIH combined with effective site-specific immunotherapies could lead to long-term remission without blanket immunosuppression and with minimal deleterious side effects.

## Introduction

Autoimmune hepatitis (AIH) is a disease of unknown etiology and, like most autoimmune diseases, is a multifactorial process involving genetic susceptibilities, dysregulation of immune tolerance mechanisms, and environmental triggers (1, 2). As in many autoimmune diseases, female subjects are more frequently affected (1, 2).

Two types of AIH have been described according to the type of circulating autoantibodies. Type 1 AIH is characterized by the presence of antismooth muscle antibodies and/or antinuclear antibodies (2). Type 2 AIH is defined by the detection of liver–kidney microsomal antibody type 1 (LKM1) (3–6) and/or liver-cytosol antibody type 1 (LC1) (7). These latter autoantibodies are directed against the cytochrome P450 2D6 (CYP2D6) (8–10) and the formiminotransferase-cyclodeaminase (FTCD) (7), respectively. Thirty percent of patients with type 2 AIH are anti-LC1 positive; in 10% of cases, anti-LC1 is the only serological marker present (7, 11, 12). Type 2 AIH is more frequent in the pediatric population than in the adult population (1, 2). Pediatric patients with type 2 AIH are often younger than patients with type 1 (1). However, both types respond equally well to current treatments (1, 2).

Conventional therapy for AIH patients consists of immunosuppressive drugs, usually a combination of prednisone/prednisolone and azathioprine to induce remission of liver inflammation (1, 2). Cyclosporine A has also been used successfully in children with AIH to induce and maintain remission (13, 14). Tacrolimus and mycophenolate mofetil can also be used but mostly in those with poor response or poor tolerance to conventional treatment (1). Recently, budesonide has been used successfully in both adults and children with AIH (15, 16). Although it results in fewer side effects such as weight gain, it is less effective than prednisone in inducing remission in children (15).

Most patients treated with current therapies show long-term complete response to treatment, but progression toward cirrhosis and end-stage liver disease occurs in 10–20% of patients and liver transplantation may be necessary (1, 3, 17). No clinical, laboratory, or histological features can accurately predict initial complete response or long-term remission (1, 18–20). Current treatments, although effective, are associated with deleterious side effects either specific to each drug or as the result of broad immunosuppression (1, 2). Treatment withdrawal is difficult to achieve with up to 90% relapse rate (21). Recently, careful patient selection based on treatment duration and liver biochemistry parameters has been reported to improve relapse rates although these remain high (22). AIH patients respond well to treatment; however, most patients will remain under lifelong immunosuppressive therapy.

Therefore, future immunotherapies should aim to restore self-tolerance to hepatic autoantigens, abrogating the need for long-term immunosuppression with its associated adverse effects. This is especially important for pediatric AIH patients for whom lifelong broad immunosuppression will lead to increased risk of adverse effects.

## Immunotherapies

Current treatments for AIH are based on non-specific immunosuppressive therapies, and although development of new specific biological immunotherapies have seen great progress in other autoimmune and inflammatory diseases, very little progress has been made in the past decades for the treatment of AIH (2). New therapies targeting specific immune cell subpopulations or cytokines could provide an effective mean of inducing rapid and complete remission in patients with AIH and minimize deleterious side effects. However, the development of such targeted therapies requires an understanding of the immune cell subsets and mediators of inflammation involved in the pathogenesis of autoimmune liver injury in AIH.

### T Lymphocytes

Autoimmune hepatitis is considered a T cell-mediated disease; liver biopsies of AIH patients show lymphoplasmacytoid infiltrates with lobular inflammation and bridging necrosis. Analysis of liver inflammatory infiltrates from AIH patients shows that most of them are composed of CD4^+^ T lymphocytes with a Th1 phenotype (23). The involvement of CD4^+^ T cells in AIH is consistent with the observation that autoantibodies found in AIH are immunoglobulin G (IgG) implying a CD4^+^ T-cell-dependent isotype class switching. In addition, CYP2D6-specific CD4^+^ T cells can be isolated from type 2 AIH patients, the same autoantigen targeted by LKM1 autoantibody-producing B cells (23). Furthermore, there is an overlap between CYP2D6 peptide sequences inducing the T- and B-cell autoimmune responses in type 2 AIH, highlighting the link between the T and B cell responses in AIH pathogenesis (24).

Although liver inflammatory infiltrates are mainly composed of CD4^+^ T cells, CD8^+^ T cells are found at the interface between the liver lobule and the portal tract and are considered responsible for hepatocyte injury (25). The cell-to-cell cytotoxic effect of CD8^+^ T cells can be mediated through either Fas/FasL (26–28), perforin/granzyme pathway (29), TNF receptors (30), or TRAIL receptors (31). Liver injury can also result from a bystander effect induced by local IFN-γ and TNF-α secretion from activated T cells (32). This non-specific damage can result in autoantigens unmasking, normally hidden from the immune system, thus amplifying the inflammation and immune-mediated liver damage. The cytotoxic activity of CD8^+^ T cells, resulting in hepatocyte death, is believed to be the end result of complex interactions between B and T cells. Therefore, targeting T cells using depleting anti-CD3 antibodies could suppress the T cell-mediated cytotoxicity against hepatocytes and possibly lead to the elimination of autoreactive CD4^+^ and CD8^+^ T cells in these patients.

A murine model of type 2 AIH has been developed based on xenoimmunization with human type 2 autoantigens (CYP2D6 and FTCD). This model replicates most clinical and laboratory characteristics of type 2 AIH, such as elevated serum ALT levels, liver inflammatory infiltrate that composed of CD4^+^, CD8^+^ T and B lymphocytes, a Th1 phenotype of autoimmune CD4^+^ T cell response, elevated immunoglobulin levels, and anti-LKM1 and anti-LC1 autoantibodies (33–37). In addition, as in humans, females are more susceptible to AIH, and development of the disease is influenced by MHC and non-MHC genes (34, 36).

**Figure 1:**
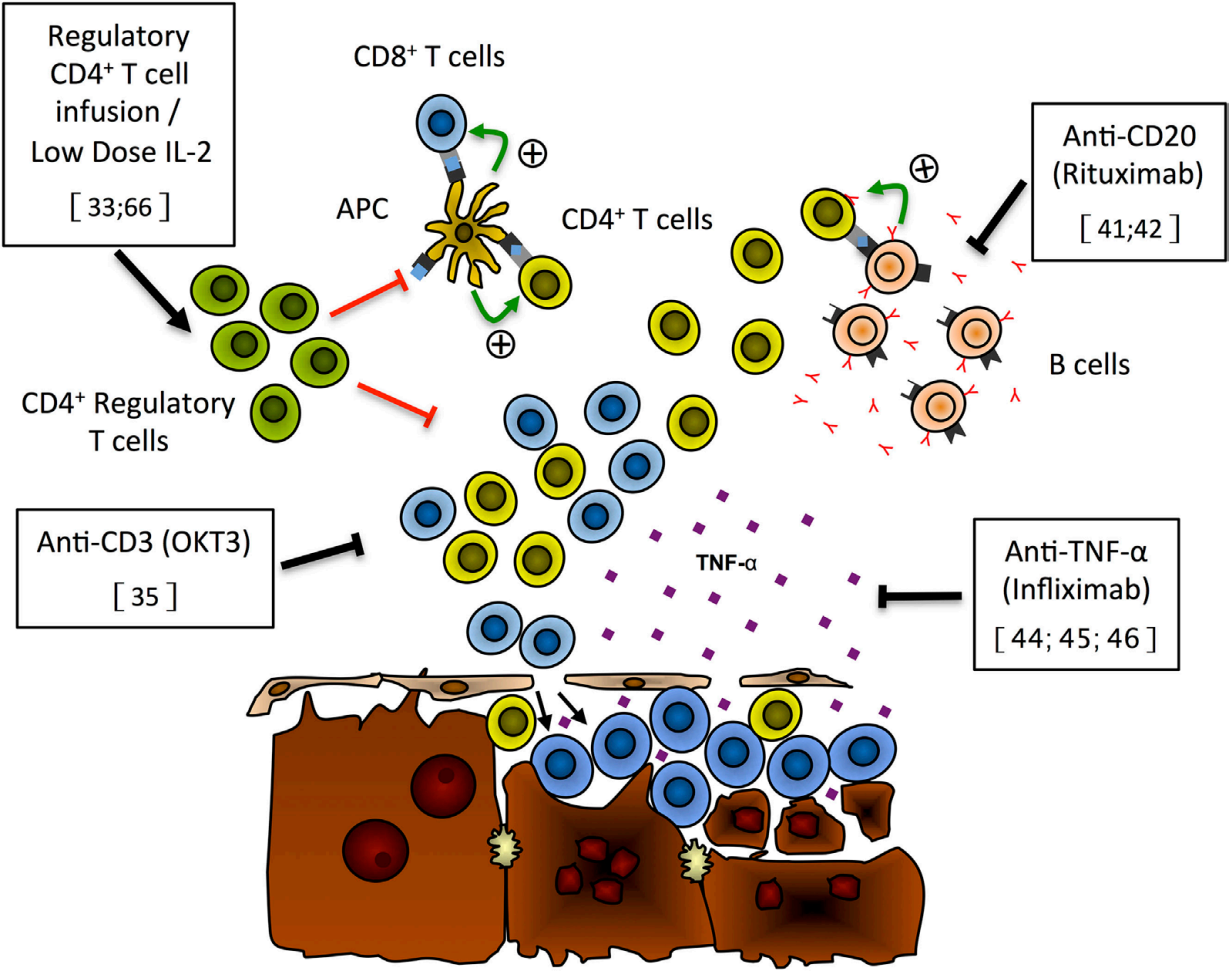
**Putative mechanisms of liver damage and new immunotherapies for autoimmune hepatitis**. On the basis of recently published data, we propose the following model for liver damage and mode of action of new immunotherapies that have shown effectiveness either in preclinical models or in preliminary patients’ reports. Expansion of the regulatory CD4^+^ T cell population (green), through Treg infusion or low-dose IL-2 treatment, can repress CD4^+^ (yellow) and CD8^+^ (blue) T cell activation and/or expansion and prevent hepatocyte lysis (33, 66). Anti-CD3 monoclonal antibodies (OKT3) can selectively deplete T cells and limit the destruction of hepatocytes (35). Selective B-cell depletion using anti-CD20 antibodies (rituximab) has the ability to prevent antigen presentation by autoreactive B cells (pink) to T cells, limiting the expansion of autoreactive T cells hence reducing hepatocyte lysis by these cells (41, 42). TNF-α blockade with monoclonal antibodies (infliximab) has been shown to reduce liver damage and induce remission of AIH (44–46).

In this model of type 2 AIH, T cell depletion using low-dose anti-CD3 antibodies was performed as a mean to induce remission (Figure 1) (35). The treatment, which reduces the number of circulating T lymphocytes by 50%, led to the disappearance of liver inflammatory infiltrates, normalization of serum aminotransferase levels, and reduced autoantibodies titers (35). In addition, residual liver-infiltrating T lymphocytes were no longer responsive to autoantigen stimulation, suggesting that these lymphocytes had been tolerized (35). These data suggest that partial T cell depletion could lead to the restoration of tolerance to hepatic autoantigens. However, more work is needed as only a single administration of anti-CD3 was performed, which, while leading to temporary remission of active AIH, did not confer long-term remission (35).

Anti-CD3 T cell-depleting monoclonal antibodies (OKT3) are used in the treatment of severe acute rejection after solid organ transplantation. Anti-CD3 treatment has been found effective in patients with type 1 diabetes (38). Its successful use in this T cell-mediated autoimmune disease and the encouraging results obtained in the experimental model of type 2 AIH (35, 38) warrant further investigation into this type of treatment. Depletion of a larger number of T cells or over a longer period of time may allow the elimination of autoreactive T cells responsible for the autoimmune liver injury, hence restoring T cell tolerance to hepatic autoantigens and leading to long-term remission. However, side effects will need to be carefully monitored since T-cell depletion mediated by anti-CD3 relies on an activated cell death mechanism that can lead to the release of cytokines such as IFN-γ and TNF-α. This can cause a cytokine release syndrome with its associated well-recognized toxicity (39). Further research is needed to evaluate if the benefits for patients with AIH outweigh the potential serious side effects.

### B Lymphocytes

Specific autoantibodies, a hallmark of AIH, are important markers for diagnosis, but their role in the pathogenesis of AIH remains controversial. In type 2 AIH, contrary to type 1, the liver autoantigens targeted by the autoantibodies are known. Theses autoantigens, targeted by anti-LKM1 and anti-LC1 autoantibodies, CYP2D6 and FTCD, are intracellular proteins expressed at low levels. While being mainly found in hepatocytes, they are not organ specific. Therefore, there are no obvious reasons to explain why these proteins are specifically targeted in AIH. In addition, there is no evidence that these specific autoantibodies directly mediate the autoimmune response against hepatocytes. However, the autoreactive polyclonal B cells found in every AIH patients could provide a population of activated professional antigen-presenting cells (APC) that could efficiently present self-peptides to naive T cells and perpetuate the autoimmune T cell reactivity against hepatocytes.

In an experimental model of type 2 AIH, the administration of a single dose of B-cell-depleting anti-CD20 antibodies resulted in a significant reduction in liver inflammation, ALT levels, and pro-inflammatory IP10 chemokine expression (Figure 1) (40). However, total IgG levels and autoantibodies were not affected by this therapy (40). There were significantly more naïve and fewer antigen-experienced CD4^+^ and CD8^+^ T cells, and T-cell proliferation was significantly reduced following anti-CD20 treatment. In this model, CD19^+^ B cells served as efficient APCs to CD4^+^ T cells, and anti-CD20 depletion of B cells led to reduced CD4^+^ T cell proliferation and reduced expression of MHC class II and CD80 by CD11b^+^ APCs and by remaining CD19^+^ B cells (40). Anti-CD20 treatment also led to a profound reduction in T follicular helper cells (40).

In adult and pediatric AIH patients, B-cell-depleting anti-CD20 antibodies (rituximab) have been used successfully in difficult-to-treat patients (41, 42). Complete remission has been achieved and maintained using rituximab, alone or in combination with standard therapy, without serious adverse effects (41, 42). Although encouraging, these reports are based on limited numbers of patients, and larger scale studies are needed to test the effectiveness of this specific immunotherapy in AIH patients. While rituximab may not replace the current standard therapy, it may prove useful in specific cases or to control disease flare-up (41).

### Cytokine Neutralization

Monoclonal antibody-mediated neutralization of cytokines has rarely been used in the treatment of AIH patients likely in part due to its complex pathogenesis and the difficulty in identifying a single mediator of liver inflammation to neutralize. TNF-α neutralization (infliximab) has been used successfully in several inflammatory pathologies including rheumatoid arthritis, psoriasis arthritis, ulcerative colitis, and Crohn’s disease (43). Infliximab has recently been used for the treatment of difficult-to-treat AIH patient as a rescue therapy, including a case of pediatric AIH (Figure 1) (44–46). In a series of 11 patients treated with anti-TNF-α, because they did not respond to standard treatment, were intolerant to azathioprine, or developed severe side effects from standard therapy, the authors reported induction of remission in 60% of cases (44). However, 4 of 11 patients developed severe infections, some requiring hospitalization, and infliximab treatment had to be stopped (44). Patients with difficult-to-treat AIH are generally at a higher risk of infectious complications following intense immunosuppressive treatment compared to those responding to standard treatment, and the presence of liver cirrhosis, as in most of these patients, increases the risk for infections (47).

These results suggest that TNF-α may have a significant role in the autoimmune liver injury present in some patients. However, its use as a rescue treatment must be carefully considered in view of the potential serious infectious side effects already reported (44). In addition, there have been several recent reports of anti-TNF-α-induced AIH in patients treated for inflammatory bowel disease, rheumatoid arthritis, or psoriasis (48–50). Therefore, further research is needed to better identify the role of TNF-α in the pathogenesis of AIH. The identification of specific biomarkers linked to TNF-α activity in AIH could allow the selection of patients who would benefit the most from anti-TNF-α-based therapy.

### Regulatory T Cells

Tregs are critical to maintain immunological tolerance against self: furthermore, Treg deficiency leads to the development of autoimmune diseases (51). Low numbers or decreased functionality of CD4^+^ Tregs has been reported in patients with AIH (52–57). However, normal frequency and functionality of FOXP3^+^ Tregs have also been reported (58). These contradictory reports may stem in part from difficulties to effectively identify human Tregs based on the markers such as CD25 and FoxP3 that can be transiently expressed by activated effector T cells (59). In addition, immunosuppressive treatment can also influence Treg levels (58). Treg frequencies in adult AIH patients under treatment are significantly reduced compared to both untreated AIH patients and healthy subjects (58). Moreover, expression levels of CD25 can be associated with disease activity in AIH patients (58). Recently, a decrease in frequency and a functional impairment of CD39^+^ Tregs have been described in AIH patients (54). CD39^+^ Tregs show preferential suppression over CD4^+^ Th17 immunity, and decreased numbers of these Tregs have also been described in patients with multiple sclerosis (60).

In an experimental model of type 2 AIH, CD4^+^ Tregs have been found to influence the outcome of the disease (33). The susceptibility of a mice strain (C57BL/6) to AIH was found to originate from their inability to expand Tregs following exposure to human antigens (33). Resistant mice strain 129S/v developed significantly higher numbers of Tregs that prevented the development of AIH. However, Tregs of the susceptible strain (C57BL/6) were fully functional (33). This suggests that the susceptible mice strain did not develop AIH due to a functional impairment of Tregs but because of the lack of Tregs. Interestingly, CXCR3^+^ Tregs from mice with AIH could be isolated, expanded *ex vivo*, and maintained their functionality (33). Adoptive transfer of these *ex vivo* expanded CXCR3^+^ Tregs in mice with AIH efficiently targeted the liver that expressed cognate ligands CXCL9 and CXCL10. This influx of CXCR3^+^ regulatory T cells to the liver restored peripheral tolerance to liver autoantigens and induced remission of AIH (Figure 1) (33).

Based on these observations, infusion of autologous *ex vivo*-expanded Tregs could be an effective therapeutic approach for the treatment of patients with AIH. This idea has generated great enthusiasm as it could lead to long-term tolerance to hepatic autoantigens (61). Efforts are currently underway to expand Tregs for infusion in type 2 AIH patients, including antigen-specific Tregs (62–64). Interestingly, Treg recruitment through the CXCR3 pathway is functional in AIH patients. Therefore, CXCR3^+^ Tregs could be used to target the inflamed liver, potentiating the effectiveness of autologous Treg infusions (65).

It is also possible to expand CD4^+^ regulatory T cells *in vivo* using low-dose IL-2 injections (66). IL-2 is a growth factor for T cells, but it preferentially expands CD4^+^ regulatory T cells due to their high levels of CD25, the IL-2 high-affinity receptor (66). Low-dose IL-2 therapy has been used successfully in patients with HCV-induced vasculitis, leading to increased numbers of circulating Tregs without adverse effects (67). However, since IL-2 can also expand effector T cells, further research is needed to understand their impact on the regulator/effector T cell balance and on the evolution of the disease in view of the large numbers of effector T cells present during an AIH.

## Long-Term Risks Associated with Immunosupression

Long-term immunosuppression is associated with an increased risk of cancer. This is particularly true in transplant patients in whom the total exposure to immunosuppressive agents has been shown to increase the risk of developing cancer (68). The type of cancers arising in this population depends on the number of factors such as age, presence of chronic infections, lifestyle, and the underlying disease. The main types of cancer found in this population are non-melanoma skin cancer and non-Hodgkin lymphomas.

Liver cell cancer, also called hepatocellular carcinoma (HCC), is a known complication of almost all chronic liver disease patients especially those with underlying cirrhosis. Indeed, the presence of cirrhosis is known to be a major determinant in the risk of developing HCC (69, 70). In patients with AIH, HCC occurs in approximately 4% of patients with a 10-year risk of 2.9% (2). In a long-term follow up of 634 Swedish patients with AIH, 4% of cirrhotic patients developed HCC with an incidence rate of 0.3% per year (71). In another study consisting of 243 patients with AIH, 12% of cirrhotic patients developed HCC with an incidence rate of 1.1% per year. Finally, in a series of 322 patients with AIH, the risk of developing HCC among cirrhotic patients was 1.9% (72, 73). In the last two series, the yearly risk is close to or above the AASLD recommended threshold of 1.5% that meets cost-effectiveness ratio for HCC monitoring (74).

These incidence rates are not as high as those found for patients with other types of liver diseases (74). However, it has been suggested that the longevity of patients with AIH-associated cirrhosis and the chronic need for immune-modifying medications may increase their risk of HCC (2).

To our knowledge, there is no report of HCC developing in AIH patients without underlying cirrhosis. This is surprising since a large epidemiological study found that cirrhosis was only diagnosed in 22% of patients with HCC who otherwise had evidence of risk factors for chronic liver disease (75). HCC has been reported in patients following kidney transplantation in absence of cirrhosis and viral hepatitis (71). Furthermore, reports have described cases of HCC in patients receiving anti-TNF therapy without liver cirrhosis (76, 77). HCC has also been reported in a patient with common variable immunodeficiency in the absence of cirrhosis (78).

One of the strongest evidence that immunosuppression increases the risk of developing HCC comes from the studies of large cohorts of HIV/HCV co-infected individuals in whom low levels of CD4^+^ T cells are linked with a risk of HCC (79, 80). Altogether one needs to be aware of the risk of HCC in AIH cirrhotic patients and the potential implications of immunosuppression in modulating this risk.

Most non-hepatic malignancies developing during chronic immunosuppression are non-melanoma skin cancers. Although rarely life threatening, they can represent a significant management challenge and lead to repeated and sometimes mutilating surgeries for patients (81). As stated earlier, chronic immunosuppression is also associated with an increased risk of developing non-Hodgkin lymphomas. Recently, two cases of hepatosplenic lymphoma have been described in adolescents treated for AIH (82). A case of immunodeficiency-associated lymphoproliferative disease has also been described in a patient receiving mycophenolate for the control of AIH (83). It is important to note that lymphoproliferative disorders are often associated with Epstein–Barr infection in immunocompromised individuals (84).

If new immunotherapies, such as infliximab and rituximab, are to be considered for the treatment of AIH, they have to show a safety profile equivalent or improved compared to current therapies. In a pooled analysis of the risk associated with the treatment of inflammatory bowel disease with infliximab, no increase in the incidence of infection, mortality, or malignancy was found compared to the placebo control group (85). In a study on the risk of malignancies in 186 rituximab-treated rheumatoid arthritis patients, the use of rituximab did not increase the risk of cancer (86). Therefore, the safety profiles of rituximab and infliximab do not seem to preclude their preliminary use for the treatment of AIH, but studies of their safety profile in AIH patients will need to be performed. Use of these immunotherapies could also have the added benefit of limiting the lifetime risk of pediatric patients to adverse events since they have the potential to restore tolerance to hepatic autoantigens and induce long-term remission, thereby minimizing the use of immunosuppression in these patients.

## Conclusion and Future Perspectives

A better understanding of the pathogenesis of AIH will likely reveal new pathways and molecular/cellular targets that could be efficiently used for treatment. In addition, better knowledge of immunological tolerance and autoimmunity may also open new therapeutic avenues. For example, a promising new experimental therapy is currently being tested in an animal model of type 2 AIH that consists of an antigen-specific intranasal desensitization that can lead to the restoration of immunological tolerance to type 2 AIH autoantigens and remission of liver inflammation (87). This type of therapy is of course dependent on our knowledge of targeted autoantigens and therefore confined, for the time being at least, to the treatment of type 2 AIH. However, this type of antigen-specific immunotherapy could do away with blanket immunosuppression, as is currently standard care in AIH patients.

The search for a magic bullet for the treatment of AIH will likely prove elusive. However, development of specific immunotherapies in combination with a better understanding of this complex disease, including the identification of specific biomarkers, will provide a broader arsenal of treatments tailored for use in selected patients. The variable response to treatment by patients with AIH is a testament to the complexity and likely heterogeneous nature of this disease. A better understanding of key molecular effectors in AIH combined with effective site-specific immunotherapies will likely be the most efficient way to induce long-term remission with minimal deleterious side effects.

## Author Contributions

All authors have made substantial contribution to the paper: SC and PL wrote the manuscript; MB and CV revised the article critically and added important intellectual content. All authors have read and approved the final version of the paper.

## Conflict of Interest Statement

The authors declare that the research was conducted in the absence of any commercial or financial relationships that could be construed as a potential conflict of interest.

## References

[1] Jimenez-Rivera C Ling SC Ahmed N Yap J Aglipay M Barrowman N Incidence and characteristics of autoimmune hepatitis. Pediatrics (2015) 136:e1237–48. 10.1542/peds.2015-0578 26482664

[2] Manns MP Czaja AJ Gorham JD Krawitt EL Mieli-Vergani G Vergani D Diagnosis and management of autoimmune hepatitis. Hepatology (2010) 51:2193–213. 10.1002/hep.23584 20513004

[3] Alvarez F Autoimmune hepatitis. In: Suchy F Sokol R Baliestreri W, editors. Liver Disease in Childhood. Philadelphia: Lippincott Williams & Wilkins (2001). p. 429–41.

[4] Homberg JC Abuaf N Bernard O Islam S Alvarez F Khalil SH Chronic active hepatitis associated with antiliver/kidney microsome antibody type 1: a second type of “autoimmune” hepatitis. Hepatology (1987) 7:1333–9. 10.1002/hep.1840070626 3679093

[5] Maggiore G Bernard O Homberg JC Hadchouel M Alvarez F Hadchouel P Liver disease associated with anti-liver-kidney microsome antibody in children. J Pediatr (1986) 108:399–404. 10.1016/S0022-3476(86)80880-0 3950819

[6] Maggiore G Veber F Bernard O Hadchouel M Homberg JC Alvarez F Autoimmune hepatitis associated with anti-actin antibodies in children and adolescents. J Pediatr Gastroenterol Nutr (1993) 17:376–81. 10.1097/00005176-199311000-00007 8145091

[7] Lapierre P Hajoui O Homberg JC Alvarez F. Formiminotransferase cyclodeaminase is an organ-specific autoantigen recognized by sera of patients with autoimmune hepatitis. Gastroenterology (1999) 116:643–9. 10.1016/S0016-5085(99)70186-1 10029623

[8] Gueguen M Boniface O Bernard O Clerc F Cartwright T Alvarez F. Identification of the main epitope on human cytochrome P450 IID6 recognized by anti-liver kidney microsome antibody. J Autoimmun (1991) 4:607–15. 10.1016/0896-8411(91)90180-K 1723273

[9] Yamamoto AM Cresteil D Boniface O Clerc FF Alvarez F. Identification and analysis of cytochrome P450IID6 antigenic sites recognized by anti-liver-kidney microsome type-1 antibodies (LKM1). Eur J Immunol (1993) 23:1105–11. 10.1002/eji.1830230519 7682958

[10] Manns MP Johnson EF Griffin KJ Tan EM Sullivan KF. Major antigen of liver kidney microsomal autoantibodies in idiopathic autoimmune hepatitis is cytochrome P450db1. J Clin Invest (1989) 83:1066–72. 10.1172/JCI113949 2466049 PMC303785

[11] Martini E Abuaf N Cavalli F Durand V Johanet C Homberg JC. Antibody to liver cytosol (anti-LC1) in patients with autoimmune chronic active hepatitis type 2. Hepatology (1988) 8:1662–6. 10.1002/hep.1840080632 3192182

[12] Abuaf N Johanet C Chretien P Martini E Soulier E Laperche S Characterization of the liver cytosol antigen type 1 reacting with autoantibodies in chronic active hepatitis. Hepatology (1992) 16:892–8. 10.1002/hep.1840160407 1398495

[13] Alvarez F Ciocca M Canero-Velasco C Ramonet M de Davila MT Cuarterolo M Short-term cyclosporine induces a remission of autoimmune hepatitis in children. J Hepatol (1999) 30:222–7. 10.1016/S0168-8278(99)80065-8 10068099

[14] Cuarterolo M Ciocca M Velasco CC Ramonet M Gonzalez T Lopez S Follow-up of children with autoimmune hepatitis treated with cyclosporine. J Pediatr Gastroenterol Nutr (2006) 43:635–9. 10.1097/01.mpg.0000235975.75120.38 17130741

[15] Woynarowski M Nemeth A Baruch Y Koletzko S Melter M Rodeck B Budesonide versus prednisone with azathioprine for the treatment of autoimmune hepatitis in children and adolescents. J Pediatr (2013) 163:1347.e–53.e. 10.1016/j.jpeds.2013.05.042 23810723

[16] Manns MP Woynarowski M Kreisel W Lurie Y Rust C Zuckerman E Budesonide induces remission more effectively than prednisone in a controlled trial of patients with autoimmune hepatitis. Gastroenterology (2010) 139:1198–206. 10.1053/j.gastro.2010.06.046 20600032

[17] Krawitt EL Autoimmune hepatitis. N Engl J Med (1996) 334:897–903. 10.1056/NEJM199604043341406 8596574

[18] Cook GC Mulligan R Sherlock S Controlled prospective trial of corticosteroid therapy in active chronic hepatitis. Q J Med (1971) 40:159–85. 10.1093/oxfordjournals.qjmed.a067264 4933363

[19] Hegarty JE Nouri Aria KT Portmann B Eddleston AL Williams R. Relapse following treatment withdrawal in patients with autoimmune chronic active hepatitis. Hepatology (1983) 3:685–9. 10.1002/hep.1840030510 6618435

[20] Kanzler S Gerken G Lohr H Galle PR Meyer zum Buschenfelde KH Lohse AW Duration of immunosuppressive therapy in autoimmune hepatitis. J Hepatol (2001) 34:354–5. 10.1016/S0168-8278(00)00095-7 11281572

[21] van Gerven NM Verwer BJ Witte BI van Hoek B Coenraad MJ van Erpecum KJ Relapse is almost universal after withdrawal of immunosuppressive medication in patients with autoimmune hepatitis in remission. J Hepatol (2013) 58:141–7. 10.1016/j.jhep.2012.09.009 22989569

[22] Hartl J Ehlken H Weiler-Normann C Sebode M Kreuels B Pannicke N Patient selection based on treatment duration and liver biochemistry increases success rates after treatment withdrawal in autoimmune hepatitis. J Hepatol (2015) 62:642–6. 10.1016/j.jhep.2014.10.018 25457202

[23] Lohr HF Schlaak JF Lohse AW Bocher WO Arenz M Gerken G Autoreactive CD4+ LKM-specific and anticlonotypic T-cell responses in LKM-1 antibody-positive autoimmune hepatitis. Hepatology (1996) 24:1416–21. 10.1002/hep.510240619 8938173

[24] Ma Y Bogdanos DP Hussain MJ Underhill J Bansal S Longhi MS Polyclonal T-cell responses to cytochrome P450IID6 are associated with disease activity in autoimmune hepatitis type 2. Gastroenterology (2006) 130:868–82. 10.1053/j.gastro.2005.12.020 16530525

[25] Wen L Peakman M Lobo-Yeo A McFarlane BM Mowat AP Mieli-Vergani G T-cell-directed hepatocyte damage in autoimmune chronic active hepatitis. Lancet (1990) 336:1527–30. 10.1016/0140-6736(90)93306-A 1979365

[26] Galle PR Hofmann WJ Walczak H Schaller H Otto G Stremmel W Involvement of the CD95 (APO-1/Fas) receptor and ligand in liver damage. J Exp Med (1995) 182:1223–30. 10.1084/jem.182.5.1223 7595193 PMC2192196

[27] Schlosser SF Azzaroli F Dao T Hingorani R Nicholas Crispe I Boyer JL. Induction of murine hepatocyte death by membrane-bound CD95 (Fas/APO-1)-ligand: characterization of an in vitro system. Hepatology (2000) 32:779–85. 10.1053/jhep.2000.18422 11003622

[28] Liu ZX Govindarajan S Okamoto S Dennert G Fas- and tumor necrosis factor receptor 1-dependent but not perforin-dependent pathways cause injury in livers infected with an adenovirus construct in mice. Hepatology (2000) 31:665–73. 10.1002/hep.510310317 10706557

[29] Adachi K Tsutsui H Kashiwamura S Seki E Nakano H Takeuchi O Plasmodium berghei infection in mice induces liver injury by an IL-12- and toll-like receptor/myeloid differentiation factor 88-dependent mechanism. J Immunol (2001) 167:5928–34. 10.4049/jimmunol.167.10.5928 11698470

[30] Abougergi MS Gidner SJ Spady DK Miller BC Thiele DL Fas and TNFR1, but not cytolytic granule-dependent mechanisms, mediate clearance of murine liver adenoviral infection. Hepatology (2005) 41:97–105. 10.1002/hep.20504 15619234 PMC2666068

[31] Zender L Hutker S Mundt B Waltemathe M Klein C Trautwein C NFkappaB-mediated upregulation of bcl-xl restrains TRAIL-mediated apoptosis in murine viral hepatitis. Hepatology (2005) 41:280–8. 10.1002/hep.20566 15660391

[32] Bowen DG Warren A Davis T Hoffmann MW McCaughan GW Fazekas de St Groth B Cytokine-dependent bystander hepatitis due to intrahepatic murine CD8 T-cell activation by bone marrow-derived cells. Gastroenterology (2002) 123:1252–64. 10.1053/gast.2002.36058 12360486

[33] Lapierre P Beland K Yang R Alvarez F. Adoptive transfer of ex vivo expanded regulatory T cells in an autoimmune hepatitis murine model restores peripheral tolerance. Hepatology (2013) 57:217–27. 10.1002/hep.26023 22911361

[34] Lapierre P Beland K Martin C Alvarez F Jr Alvarez F. Forkhead box p3+ regulatory T cell underlies male resistance to experimental type 2 autoimmune hepatitis. Hepatology (2010) 51:1789–98. 10.1002/hep.23536 20232291

[35] Marceau G Yang R Lapierre P Beland K Alvarez F Low-dose anti-CD3 antibody induces remission of active autoimmune hepatitis in xenoimmunized mice. Liver Int (2015) 35:275–84. 10.1111/liv.12498 24517723

[36] Lapierre P Beland K Djilali-Saiah D Alvarez F. Type 2 autoimmune hepatitis murine model: the influence of genetic background in disease development. J Autoimmun (2006) 26:82–9. 10.1016/j.jaut.2005.11.001 16380229

[37] Lapierre P Djilali-Saiah I Vitozzi S Alvarez F. A murine model of type 2 autoimmune hepatitis: xenoimmunization with human antigens. Hepatology (2004) 39:1066–74. 10.1002/hep.20109 15057911

[38] Herold KC Hagopian W Auger JA Poumian-Ruiz E Taylor L Donaldson D Anti-CD3 monoclonal antibody in new-onset type 1 diabetes mellitus. N Engl J Med (2002) 346:1692–8. 10.1056/NEJMoa012864 12037148

[39] Maude SL Barrett D Teachey DT Grupp SA. Managing cytokine release syndrome associated with novel T cell-engaging therapies. Cancer J (2014) 20:119–22. 10.1097/PPO.0000000000000035 24667956 PMC4119809

[40] Beland K Marceau G Labardy A Bourbonnais S Alvarez F. Depletion of B cells induces remission of autoimmune hepatitis in mice through reduced antigen presentation and help to T cells. Hepatology (2015) 62:1511–23. 10.1002/hep.27991 26175263

[41] D’Agostino D Costaguta A Alvarez F. Successful treatment of refractory autoimmune hepatitis with rituximab. Pediatrics (2013) 132:e526–30. 10.1542/peds.2011-1900 23821693

[42] Burak KW Swain MG Santodomingo-Garzon T Lee SS Urbanski SJ Aspinall AI Rituximab for the treatment of patients with autoimmune hepatitis who are refractory or intolerant to standard therapy. Can J Gastroenterol (2013) 27:273–80. 10.1155/2013/512624 23712302 PMC3735730

[43] Karampetsou MP Liossis SN Sfikakis PP TNF-alpha antagonists beyond approved indications: stories of success and prospects for the future. QJM (2010) 103:917–28. 10.1093/qjmed/hcq152 20802008

[44] Weiler-Normann C Schramm C Quaas A Wiegard C Glaubke C Pannicke N Infliximab as a rescue treatment in difficult-to-treat autoimmune hepatitis. J Hepatol (2013) 58:529–34. 10.1016/j.jhep.2012.11.010 23178709

[45] Rajanayagam J Lewindon PJ Infliximab as rescue therapy in paediatric autoimmune hepatitis. J Hepatol (2013) 59:908–9. 10.1016/j.jhep.2013.05.046 23792030

[46] Weiler-Normann C Wiegard C Schramm C Lohse AW A case of difficult-to-treat autoimmune hepatitis successfully managed by TNF-alpha blockade. Am J Gastroenterol (2009) 104:2877–8. 10.1038/ajg.2009.433 19888264

[47] Tandon P Garcia-Tsao G. Bacterial infections, sepsis, and multiorgan failure in cirrhosis. Semin Liver Dis (2008) 28:26–42. 10.1055/s-2008-1040319 18293275

[48] Rodrigues S Lopes S Magro F Cardoso H Horta e Vale AM Marques M Autoimmune hepatitis and anti-tumor necrosis factor alpha therapy: a single center report of 8 cases. World J Gastroenterol (2015) 21:7584–8. 10.3748/wjg.v21.i24.7584 26140007 PMC4481456

[49] van Casteren-Messidoro C Prins G van Tilburg A Zelinkova Z Schouten J de Man R Autoimmune hepatitis following treatment with infliximab for inflammatory bowel disease. J Crohns Colitis (2012) 6:630–1. 10.1016/j.crohns.2012.01.017 22398075

[50] Dang LJ Lubel JS Gunatheesan S Hosking P Su J. Drug-induced lupus and autoimmune hepatitis secondary to infliximab for psoriasis. Australas J Dermatol (2014) 55:75–9. 10.1111/ajd.12054 23651182

[51] Sakaguchi S Yamaguchi T Nomura T Ono M. Regulatory T cells and immune tolerance. Cell (2008) 133:775–87. 10.1016/j.cell.2008.05.009 18510923

[52] Liberal R Grant CR Ma Y Csizmadia E Jiang ZG Heneghan MA CD39 mediated regulation of Th17-cell effector function is impaired in juvenile autoimmune liver disease. J Autoimmun (2016) 72:102–12. 10.1016/j.jaut.2016.05.005 27210814 PMC6348153

[53] Liberal R Grant CR Holder BS Cardone J Martinez-Llordella M Ma Y In autoimmune hepatitis type 1 or the autoimmune hepatitis-sclerosing cholangitis variant defective regulatory T-cell responsiveness to IL-2 results in low IL-10 production and impaired suppression. Hepatology (2015) 62:863–75. 10.1002/hep.27884 25953611

[54] Grant CR Liberal R Holder BS Cardone J Ma Y Robson SC Dysfunctional CD39(POS) regulatory T cells and aberrant control of T-helper type 17 cells in autoimmune hepatitis. Hepatology (2014) 59:1007–15. 10.1002/hep.26583 23787765 PMC6377365

[55] Ferri S Longhi MS De Molo C Lalanne C Muratori P Granito A A multifaceted imbalance of T cells with regulatory function characterizes type 1 autoimmune hepatitis. Hepatology (2010) 52:999–1007. 10.1002/hep.23792 20683931

[56] Longhi MS Hussain MJ Mitry RR Arora SK Mieli-Vergani G Vergani D Functional study of CD4+CD25+ regulatory T cells in health and autoimmune hepatitis. J Immunol (2006) 176:4484–91. 10.4049/jimmunol.176.7.4484 16547287

[57] Longhi MS Ma Y Bogdanos DP Cheeseman P Mieli-Vergani G Vergani D. Impairment of CD4(+)CD25(+) regulatory T-cells in autoimmune liver disease. J Hepatol (2004) 41:31–7. 10.1016/j.jhep.2004.03.008 15246204

[58] Peiseler M Sebode M Franke B Wortmann F Schwinge D Quaas A FOXP3+ regulatory T cells in autoimmune hepatitis are fully functional and not reduced in frequency. J Hepatol (2012) 57:125–32. 10.1016/j.jhep.2012.02.029 22425700

[59] Wang J Ioan-Facsinay A van der Voort EI Huizinga TW Toes RE. Transient expression of FOXP3 in human activated nonregulatory CD4+ T cells. Eur J Immunol (2007) 37:129–38. 10.1002/eji.200636435 17154262

[60] Fletcher JM Lonergan R Costelloe L Kinsella K Moran B O’Farrelly C CD39+Foxp3+ regulatory T cells suppress pathogenic Th17 cells and are impaired in multiple sclerosis. J Immunol (2009) 183:7602–10. 10.4049/jimmunol.0901881 19917691

[61] Vierling JM Autoimmune hepatitis and antigen-specific T regulatory cells: when can we send in the regulators? Hepatology (2011) 53:385–8. 10.1002/hep.24153 21274860

[62] Longhi MS Hussain MJ Kwok WW Mieli-Vergani G Ma Y Vergani D. Autoantigen-specific regulatory T cells, a potential tool for immune-tolerance reconstitution in type-2 autoimmune hepatitis. Hepatology (2011) 53:536–47. 10.1002/hep.24039 21274874

[63] Longhi MS Liberal R Holder B Robson SC Ma Y Mieli-Vergani G Inhibition of interleukin-17 promotes differentiation of CD25(-) cells into stable T regulatory cells in patients with autoimmune hepatitis. Gastroenterology (2012) 142:1526–35.e6. 10.1053/j.gastro.2012.02.041 22387392

[64] Longhi MS Meda F Wang P Samyn M Mieli-Vergani G Vergani D Expansion and de novo generation of potentially therapeutic regulatory T cells in patients with autoimmune hepatitis. Hepatology (2008) 47:581–91. 10.1002/hep.22071 18220288

[65] Oo YH Weston CJ Lalor PF Curbishley SM Withers DR Reynolds GM Distinct roles for CCR4 and CXCR3 in the recruitment and positioning of regulatory T cells in the inflamed human liver. J Immunol (2010) 184:2886–98. 10.4049/jimmunol.0901216 20164417

[66] Klatzmann D Abbas AK. The promise of low-dose interleukin-2 therapy for autoimmune and inflammatory diseases. Nat Rev Immunol (2015) 15:283–94. 10.1038/nri3823 25882245

[67] Saadoun D Rosenzwajg M Joly F Six A Carrat F Thibault V Regulatory T-cell responses to low-dose interleukin-2 in HCV-induced vasculitis. N Engl J Med (2011) 365:2067–77. 10.1056/NEJMoa1105143 22129253

[68] Villeneuve PJ Schaubel DE Fenton SS Shepherd FA Jiang Y Mao Y. Cancer incidence among Canadian kidney transplant recipients. Am J Transplant (2007) 7:941–8. 10.1111/j.1600-6143.2007.01736.x 17331115

[69] Fattovich G Stroffolini T Zagni I Donato F. Hepatocellular carcinoma in cirrhosis: incidence and risk factors. Gastroenterology (2004) 127:S35–50. 10.1053/j.gastro.2004.09.014 15508101

[70] El-Serag HB Rudolph KL. Hepatocellular carcinoma: epidemiology and molecular carcinogenesis. Gastroenterology (2007) 132:2557–76. 10.1053/j.gastro.2007.04.061 17570226

[71] Danielsson Borssen A Almer S Prytz H Wallerstedt S Friis-Liby IL Bergquist A Hepatocellular and extrahepatic cancer in patients with autoimmune hepatitis – a long-term follow-up study in 634 Swedish patients. Scand J Gastroenterol (2015) 50:217–23. 10.3109/00365521.2014.983154 25483724

[72] Bruix J Sherman M American Association for the Study of Liver Diseases, Practice Guidelines Committee Management of hepatocellular carcinoma. Hepatology (2005) 42:1208–36. 10.1002/hep.20933 16250051

[73] Wong RJ Gish R Frederick T Bzowej N Frenette C. Development of hepatocellular carcinoma in autoimmune hepatitis patients: a case series. Dig Dis Sci (2011) 56:578–85. 10.1007/s10620-010-1444-6 21046244

[74] Bruix J Sherman M American Association for the Study of Liver Diseases Management of hepatocellular carcinoma: an update. Hepatology (2011) 53:1020–2. 10.1002/hep.24199 21374666 PMC3084991

[75] Sanyal A Poklepovic A Moyneur E Barghout V. Population-based risk factors and resource utilization for HCC: US perspective. Curr Med Res Opin (2010) 26:2183–91. 10.1185/03007995.2010.506375 20666689

[76] Kumar A Le DT Hepatocellular carcinoma regression after cessation of immunosuppressive therapy. J Clin Oncol (2016) 34:e90–2. 10.1200/JCO.2013.51.4067 25245441 PMC4886225

[77] Chen SC Cummings OW Hartley MP Filomena CA Cho WK. Hepatocellular carcinoma occurring in a patient with Crohn’s disease treated with both azathioprine and infliximab. Dig Dis Sci (2006) 51:952–5. 10.1007/s10620-005-9009-9 16670938

[78] Gandhi K Parikh P Aronow WS Desai H Amin H Sharma M A case of explosive progression of hepatocellular carcinoma in a patient with common variable immunodeficiency (CVID). J Gastrointest Cancer (2010) 41:281–4. 10.1007/s12029-010-9158-8 20473587

[79] Schmidt N Thimme R. Role of immunity in pathogenesis and treatment of hepatocellular carcinoma. Dig Dis (2016) 34:429–37. 10.1159/000444558 27170398

[80] Gjaerde LI Shepherd L Jablonowska E Lazzarin A Rougemont M Darling K Trends in incidences and risk factors for hepatocellular carcinoma and other liver events in HIV and hepatitis C virus-coinfected individuals from 2001 to 2014: a multicohort study. Clin Infect Dis (2016) 63:821–9. 10.1093/cid/ciw380 27307505 PMC4996136

[81] Bangash HK Colegio OR. Management of non-melanoma skin cancer in immunocompromised solid organ transplant recipients. Curr Treat Options Oncol (2012) 13:354–76. 10.1007/s11864-012-0195-3 22592596

[82] Brinkert F Arrenberg P Krech T Grabhorn E Lohse A Schramm C. Two cases of hepatosplenic T-cell lymphoma in adolescents treated for autoimmune hepatitis. Pediatrics (2016) 138(3). 10.1542/peds.2015-4245 27516526

[83] Adams B Lazarchick J Medina AM Willner IR Neville B Murphy E Iatrogenic immunodeficiency-associated lymphoproliferative disease of the Hodgkin lymphoma-like variant in a patient treated with mycophenolate mofetil for autoimmune hepatitis. Am J Hematol (2010) 85:627–9. 10.1002/ajh.21753 20658594

[84] Hartmann C Schuchmann M Zimmermann T. Posttransplant lymphoproliferative disease in liver transplant patients. Curr Infect Dis Rep (2011) 13:53–9. 10.1007/s11908-010-0145-9 21308455

[85] Lichtenstein GR Rutgeerts P Sandborn WJ Sands BE Diamond RH Blank M A pooled analysis of infections, malignancy, and mortality in infliximab- and immunomodulator-treated adult patients with inflammatory bowel disease. Am J Gastroenterol (2012) 107:1051–63. 10.1038/ajg.2012.89 22613901 PMC3390465

[86] Slimani S Lukas C Combe B Morel J. Rituximab in rheumatoid arthritis and the risk of malignancies: report from a French cohort. Joint Bone Spine (2011) 78:484–7. 10.1016/j.jbspin.2010.11.012 21196130

[87] Béland K Yang R Boury F Gagnon MF Marceau G Lapierre P Abstracts of the 63rd Annual Meeting of the American Association for the Study of Liver Diseases. November 9–13, 2012. Boston, Massachusetts, USA. Hepatology (2012) 56:303A 10.1002/hep.26040 23074707

